# An update on the prevalence of metabolic syndrome and its associated factors in rural northeast China

**DOI:** 10.1186/1471-2458-14-877

**Published:** 2014-08-26

**Authors:** Shasha Yu, Xiaofan Guo, Hongmei Yang, Liqiang Zheng, Yingxian Sun

**Affiliations:** Department of Cardiology, The First Hospital of China Medical University, 155 Nanjing North Street, Heping District, 110001 Shenyang, Liaoning China; Department of Clinical Epidemiology, Shenjing Hospital of China Medical University, Shenyang, Liaoning China

**Keywords:** Metabolic syndrome, Prevalence, Risk factor, Hypertension

## Abstract

**Background:**

The last study reported the prevalence of Metabolic Syndrome (MetS) in rural Northeast China was conducted approximately ten years ago. We aimed to update the data on the prevalence and epidemiological features of MetS in rural Northeast China.

**Methods:**

This survey was conducted from July 2012 to August 2013. In this study, a total of 11,496 residents from the rural Northeast China were randomly selected and examined. MetS was defined according to the ATPIII-modified criteria. Data regarding the demographic and lifestyle characteristics and the blood biochemical indexes of these participants were collected by well-trained personnel.

**Results:**

The prevalence of MetS was 39.0% and was more prevalent in women than in men (45.6% vs. 31.4%, *P <* 0.001). Elevated blood pressure was the most common metabolic disorder in both genders (71.7% in males and 63.3% in females). Only 11.2% and 9.6% of males and females, respectively, in this study had no metabolic disorders. Multivariate logistic regression, after adjusting for possible confounders, revealed the following factors that increased the risk of MetS: being female, older age, having more than one child, a family income of >20,000 CNY per year, longer sleep duration (>9 h/d), chronic disease status, frequent consumption of beans or bean products and frequent tea drinking. Completion of education through middle school, moderate physical activity and smoking were correlated with lower rates of MetS.

**Conclusion:**

The prevalence of MetS was high in the rural areas of China, especially among females. In addition to some of the more conventional risk factors associated with MetS, including age, sex, annual income and educational status, we also found that having more than one child and frequent consumption of tea and beans were risk factors for MetS, while smoking was a common factor among those that did not have MetS in rural Northeast China.

## Background

Metabolic syndrome (MetS), which is characterized by hyperlipidemia, elevated blood pressure, high fasting blood glucose and abdominal obesity, has become one of the greatest problems in the public healthcare system in both developed and developing countries. Previous studies have reported an increase in MetS in many developing countries, including the Philippines (19%), Malaysia (24.2%), India (28.8%), Turkey (33.4%), Iran (33.7%), Venezuela (31.2%) and Brazil (25.4%) [1]. Although the prevalence of MetS in China has not been extensively investigated, a relatively higher prevalence of MetS has been noted in several coastal and inland areas, including Sichuan (23.8%), Shanghai (29.34%), Beijing (23.2%) and Guangdong (26,7%) [2–4]. However, most of the studies examining the prevalence of MetS in China recruited participants from developed or urban areas. A nationwide population-based survey held in 2005 found that 57.01% of Chinese residents lived in rural areas. The prevalence of MetS is geographically varied in China [5]. People in rural areas have completely different lifestyles than those in urban areas. For example, adults in rural Northeast China prefer pickled cabbage and green Chinese onion dipped in soy sauce while those in urban areas do not.

Our previous study reported that 33.9% (men: 16.1%; women: 47.8%) of Han and 37.6% (men: 14.7%; women: 55.0%) of Mongolians had MetS, in accordance with the International Diabetes Federation definition for MetS [6]. Nevertheless, this study recruited only hypertensive residents and was performed approximately ten years ago. Rapid economic growth was seen in the rural areas of China followed by changes in dietary structure and lifestyle. We suspect that the prevalence and epidemiological features of MetS may have changed during the past ten years. Therefore, we performed this study to update the data regarding the prevalence of MetS and its risk factors in rural China in a large sample size of rural population during 2012–2013.

## Methods

### Study population

Liaoning Province is located in Northeast China. From January 2012 to August 2013, a representative sample of participants aged ≥ 35 years was selected to characterize the prevalence, incidence and natural history of cardiovascular risk factors in rural areas of Liaoning Province. The study adopted a multi-stage, stratified, random-cluster sampling scheme. In the first stage, three counties (Dawa, Zhangwu and Liaoyang County) were selected from the eastern, southern and northern regions of Liaoning province. In the second stage, one town was randomly selected from each county (for a total of three towns). In the third stage, 8–10 rural villages from each town were randomly selected (for a total of 26 rural villages). Participants who were pregnant or had malignant tumors or mental disorders were excluded from the study. All the eligible permanent residents aged ≥ 35 years from each village were invited to attend the study (a total of 14,016 participants). Of those, 11,956 participants agreed and completed the study to give a response rate of 85.3%. The study was approved by the Ethics Committee of China Medical University (Shenyang, China). All procedures were performed in accordance with ethical standards. Written consent was obtained from all participants after they had been informed of the objectives, benefits, medical items and confidentiality agreement regarding their personal information. For participants who were illiterate, we obtained written informed consent from their proxies. In this report, we used only the data from participants who completed the study, which provided a final sample size of 11,496 (5309 men and 6187 women).

### Data collection and measurements

Data were collected during a single visit to the clinic by cardiologists and trained nurses using a standard questionnaire in a face-to-face interview. Before the survey was performed, we invited all eligible investigators to attend an organized training session. The training included the purpose of this study, how to administer the questionnaire, the standard method of measurement, the importance of standardization and the study procedures. A strict test was administered after this training, and only those who scored perfectly on the test were accepted as investigators in this study. During data collection, our inspectors had further instructions and support.

Data regarding the demographic characteristics, lifestyle risk factors, dietary habits, family income and family history of chronic diseases were obtained during the interview using the standardized questionnaire. The study was guided by a central steering committee with a subcommittee for quality control. Educational level was assessed as completion of primary school or less, middle school or high school and higher. Self-reported sleep duration (including nocturnal and nap duration) was obtained from the questionnaire. The responses were categorized into four groups: ≤7, 7–8, 8–9 and >9 h/d. Family income was classified as ≤5000, 5000–20,000 and >20,000 CNY/year. The consumption of beans or bean products was assessed by the frequency eaten per week using the following scale: rarely = 0, 2–3 times = 1 and ≥ 4 times = 2. The consumption of tea was also assessed by the frequency of consumption per day) using the following scale: no = 1, rarely = 2, 1–2 times = 3 and ≥ 3 times = 4.

According to American Heart Association protocol, blood pressure (BP) was measured three times at 2-min intervals after at least 5 min of rest using a standardized automatic electronic sphygmomanometer (HEM-907; Omron), which had been validated according to the British Hypertension Society protocol [7]. The participants were advised to avoid caffeinated beverages and exercise for at least 30 min before the measurement. During the measurement, the participants were seated with their arms supported at the level of the heart. The mean of three BP measurements was calculated and used in all analyses.

Weight and height were measured to the nearest 0.1 kg and 0.1 cm, respectively, with the participants wearing light-weight clothing and without shoes. Waist circumference (WC) was measured at the umbilicus using a non-elastic tape (to the nearest 0.1 cm), with the participants standing at the end of normal expiration. Body mass index (BMI) was calculated as the weight in kilograms divided by the square root of the height in meters.

Fasting blood samples were collected in the morning after at least 12 h of fasting. Blood samples were obtained from an antecubital vein into Vacutainer tubes containing ethylenediaminetetraacetic acid (EDTA). Fasting plasma glucose (FPG), total cholesterol (TC), low-density lipoprotein cholesterol (LDL-C), high-density lipoprotein cholesterol (HDL-C), triglycerides (TGs) and other routine blood biochemical indexes were analyzed enzymatically using an autoanalyzer. All laboratory equipment was calibrated, and blinded duplicate samples were used for these analyses.

### Definitions

According to the ATPIII-modified criteria, MetS was defined by the fulfillment of three or more the following criteria: 1) WC ≥ 90 cm in males and ≥ 80 cm in females; 2) BP ≥ 130/85 mmHg or currently taking hypertension medication; 3) serum glucose level ≥ 5.6 mmol/L or currently taking diabetes medication; 4) serum TGs ≥ 1.7 mmol/L or currently taking abnormal lipid medication; and 5) HDL-C < 1.03 mmol/L in males and < 1.29 mmol/L in females [8].

Physical activity included occupational and leisure-time physical activity. A detailed description of the methods for assessing physical activity has been presented elsewhere [9]. Occupational and leisure-time physical activity were merged and regrouped into the following three categories: 1) low—subjects who reported light levels of both occupational and leisure-time physical activity, 2) moderate—subjects who reported moderate or high levels of either occupational or leisure-time physical activity and 3) high—subjects who reported a moderate or high level of both occupational and leisure-time physical activity.

Dietary patterns were assessed by having participants recall the foods they had eaten during the previous year. The questionnaire included questions regarding the average consumption of several food items per week. The reported consumption was quantified approximately in terms of grams per week. Vegetable consumption was assessed on the following scale: rarely = 3, <1000 g = 2, 1000–2000 g = 1, ≥2000 g = 0, and meat consumption, including red meat, fish and poultry was assessed on the following scale: rarely = 0, <250 g = 1, 250–500 g = 2 and ≥500 g = 3). A special diet score (vegetable consumption score plus meat consumption score) was calculated for each participant (range 0–6). Higher values of the diet score indicated higher meat consumption, lower vegetable consumption and greater adherence to a Westernized diet, while lower values indicate adherence to the Chinese diet. Similar methods for calculating a diet score can be found in the ATTICA study [10].

### Statistical analysis

Descriptive statistics were calculated for all the variables, including continuous variables (reported as mean values and standard deviations) and categorical variables (reported as numbers and percentages). The differences between the healthy and MetS groups were evaluated using the Student’s t-test, ANOVA, non-parametric test or the χ2-test, as appropriate. Multivariate logistic regression analyses were used to identify independent factors of MetS, and odds ratios (ORs) and corresponding 95% confidence intervals (CIs) were calculated. All the statistical analyses were performed using SPSS version 17.0 software, and *P* values less than 0.05 were considered to be statistically significant.

## Results

### Baseline characteristics of study population

There were 5309 men and 6187 women aged ≥ 35 years evaluated in this cross-sectional study. The mean age was 53 years. The baseline characteristics of the study subjects according to gender are shown in Table 1. The prevalence of MetS among all the study participants was 39% and was significantly higher in women (45.6%) than in men (31.4%).

**Table 1:** **Characteristics of study population by gender in the rural population of Liaoning Province,China**

Variables	Total	Men	Women	*P-*value
	(11496)	(5309)	(6187)	
**Age (year)**	53.82 ± 10.57	54.35 ± 10.79	53.37 ± 10.35	<0.001
**Ethnicity**				0.327
Han	10898(94.8)	5027(94.7)	5871(94.9)	
Others^a^	598(5.2)	282(5.3)	316(5.1)	
**Educational status**				<0.001
Primary school or below	5726(49.8)	2211(41.6)	3515(56.8)	
Middle school	4687(40.8)	2491(46.9)	2196(35.5)	
High school or above	1083(9.4)	607(11.4)	476(7.7)	
**Physical activity**				<0.001
Light	3421(29.8)	1200(22.6)	2221(35.9)	
Moderate	7429(64.6)	3816(71.9)	3613(58.4)	
Severe	646(5.6)	293(5.5)	353(5.7)	
**Annual income (CNY/year)**				0.011
≤5000	1427(12.4)	711(13.4)	716(11.6)	
5000-20000	6260(54.5)	2845(53.6)	3415(55.2)	
>20000	3809(33.1)	1753(33.0)	2056(33.2)	
**Current smoking status (Yes)**	4056(35.3)	3028(57.0)	1028(16.6)	<0.001
**Current drinking status (Yes)**	2593(22.6)	2410(45.4)	183(3.0)	<0.001
**Marriage (Yes)**	10530(91.6)	4896(92.2)	5634(91.1)	0.014
**Diet score**	2.33 ± 1.13	2.55 ± 1.10	2.14 ± 1.11	<0.001
**Sleep duration (h/d)**	7.26 ± 2.70	7.42 ± 1.63	7.12 ± 1.74	<0.001
**SBP (mmHg)**	141.76 ± 23.47	143.66 ± 22.66	140.14 ± 24.04	<0.001
**DBP (mmHg)**	82.05 ± 11.77	83.78 ± 11.83	80.57 ± 11.52	<0.001
**BMI (kg/m** ^**2**^ **)**	24.80 ± 3.66	24.73 ± 3.54	24.86 ± 3.77	0.058
**WC (cm)**	82.43 ± 9.83	83.78 ± 9.76	81.28 ± 9.74	<0.001
**TC (mmol/L)**	5.23 ± 1.08	5.16 ± 1.04	5.29 ± 1.12	<0.001
**TG (mmol/L)**	1.63 ± 1.49	1.66 ± 1.66	1.61 ± 1.33	0.123
**LDL-C (mmol/L)**	2.92 ± 0.82	2.87 ± 0.79	2.97 ± 0.84	<0.001
**HDL-C (mmol/L)**	1.41 ± 0.38	1.41 ± 0.42	1.41 ± 0.34	0.679
**FPG (mmol/L)**	5.90 ± 1.64	5.95 ± 1.67	5.86 ± 1.60	0.003
**Cardiovascular disease** ^**b**^	1742(15.2)	568(10.7)	1174(19.0)	<0.001
**Cerebrovascular disease** ^**C**^	1012(8.8)	490(9.3)	522(8.5)	0.068
**Chronic kidney disease** ^**d**^	152(1.3)	64(1.2)	88(1.4)	0.162
**Metabolic syndrome**	4488(39.0)	1668(31.4)	2820(45.6)	<0.001

Data are expressed as the mean ± SD or as n (%). *Abbreviations:* *BMI* body mass index, *WC* waist circumference, *CNY* China Yuan (1CNY = 0.161 USD), *SBP* systolic blood pressure, *DBP* diastolic blood pressure, *TC* total cholesterol, *TG* triglyceride, *LDL-C* low-density lipoprotein cholesterol, *HDL-C* high-density lipoprotein cholesterol, *FPG* fasting plasma glucose. ^a^Including some ethnic minorities in China, such as Mongol and Manchu. ^**b**^Cardiovascular disease include angina, myocardia infarction, arrhythmia, heart failure. ^**C**^Cerebrovascular disease include cerebral hemorrhage, cerebral infarction, subarachnoid hemorrhage, Transient Ischemic Attack. ^**d**^Chronic kidney disease nephritis, acute/chronic renal failure.

### Prevalence of the MetS and its criteria

**Figure 1:**
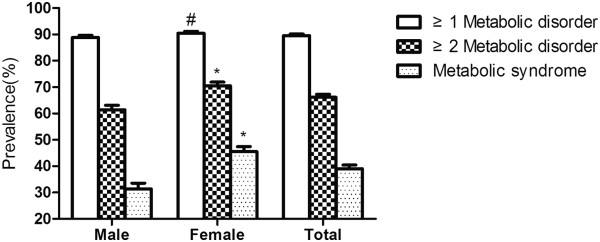
**Prevalence of the metabolic syndrome and components among rural population of Liaoning Province,China.** * *P <* 0.001 for male:female difference in proportion; ^#^ *P <* 0.05 for male:female difference in proportion.

**Figure 2:**
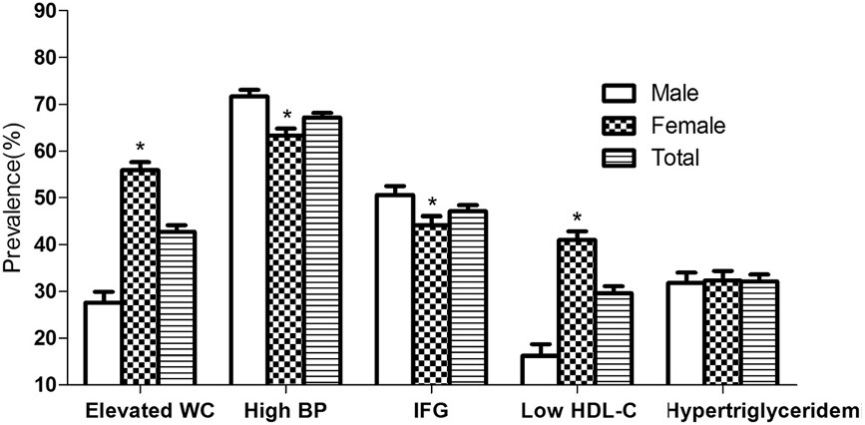
**Prevalence of the metabolic components among rural population of Liaoning Province,China.** Elevated WC:Elevated waist circumference; High BP:High Blood pressure; IFG:Increased fasting glucose; *P < 0.001 for male:female difference in proportion.

The prevalence of MetS assessed as fulfilling three or more ATPIII-modified criteria, two or more criteria, one or more criteria and the individual metabolic syndrome criteria are shown in Figures 1 and 2. The overall prevalence of the MetS was 39.0% (95% CI: 37.6%-40.4%). A total of 89.6% (95% CI: 89.0%-90.2%) participants fulfilled at least one criterion, and 66.2% (95% CI: 65.1%-67.3%) fulfilled two or more criteria. Women were more likely to have more than one or two metabolic disorders than men. Of the individual MetS criteria, high blood pressure had by far the highest prevalence, 67.2% (95% CI: 66.2%-68.2%). Increased fasting glucose had the second highest prevalence 47.1% (95% CI: 45.8%-48.4%). The prevalence of abdominal obesity in the rural residents was 42.8% (95% CI: 41.4-44.2). High TGs and low HDL-C were of relatively lower prevalence, 32.1% (95% CI: 30.6%-33.6%) and 29.6% (95% CI: 28.1%-31.1%), respectively. Impaired fasting glucose and elevated BP were more common in males, while females had higher prevalence of both abdominal obesity and low HDL.

### Prevalence of MetS based on demographics and socioeconomic status

Selected risk factor characteristics of MetS for participants in this study are presented in Table 2. The prevalence of MetS increased with age only in women. The lowest prevalence of MetS was found in the participants that had completed middle school and participated in regular moderate physical activity in either gender. The highest prevalence of MetS in females was found in those who had more than one child, an annual income ≤5000 CNY/year, slept between 8–9 h/d, were not married, did not drink alcohol, had a diet score <3 and with chronic diseases. While in men, those who were not married, were current smokers and drinkers, did not have chronic diseases and rarely consumed beans or bean products had a relatively lower risk of MetS. In all, individuals who drank 3–4 times/day had lowest risk of MetS.

**Table 2:** **Prevalence of MetS by levels of different status markers and multiple logistic regression analysis of MetS and the associated factors based on ATPIII-modified criteria in the rural population of Liaoning Province, China**

	Male			Female			Total		
Variables	N	Pre (%)	OR (95% CI)	N	Pre (%)	OR (95% CI)	N	Pre (%)	OR (95% CI)
**Age group**									
35-44	373	30.9	1.00(reference)	**411**	**26.9**	1.00(reference)	**784**	**28.7**	1.00(reference)
45-54	509	31.7	1.01(0.86,1.19)	**854**	**43.4**	**1.95(1.68,2.27)**	**1363**	**38.1**	**1.49(1.33,1.67)**
55-64	530	32.6	0.99(0.83,1.19)	**1033**	**56.3**	**2.86(2.41,3.39)**	**1563**	**45.2**	**1.81(1.60,2.05)**
≥65	873	29.3	***0.77(0.61,0.97)***	**522**	**61.1**	**3.20(2.56,3.99)**	**778**	**45.0**	**1.64(1.40,1.91)**
**Numbers of child**									
≤1	683	31.3	1.00(reference)	**917**	**37.6**	1.00(reference)	**1600**	**34.6**	1.00(reference)
>1	985	31.5	1.03(0.90,1.17)	**1903**	**50.8**	***1.20(1.06,1.35)***	**2888**	**42.0**	***1.13(1.04,1.24)***
**Ethnicity**									
Han	1588	31.6	1.00(reference)	2690	45.8	1.00(reference)	***4278***	***39.3***	1.00(reference)
Others^a^	80	28.4	0.82(0.62,1.08)	130	41.1	0.91(0.72,1.17)	***210***	***35.1***	0.87(0.73,1.05)
**Educational status**									
Primary school or below	**666**	**30.1**	1.00(reference)	**1811**	**51.5**	1.00(reference)	**2477**	**43.3**	1.00(reference)
Middle school	**765**	**30.7**	0.98(0.86,1.13)	**824**	**37.5**	***0.84(0.74,0.95)***	**1589**	**33.9**	***0.88(0.80,0.96)***
High school or above	**237**	**39.0**	***1.31(1.07,1.60)***	**185**	**38.9**	0.84(0.68,1.04)	**422**	**39.0**	1.034(0.89,1.20)
**Physical activity**									
Light	**444**	**37.0**	1.00(reference)	**1133**	**51.0**	1.00(reference)	**1577**	**46.1**	1.00(reference)
Moderate	**1121**	**29.4**	**0.75(0.65,0.87)**	**1505**	**41.7**	***0.87(0.77,0.97)***	**2626**	**35.3**	**0.81(0.74,0.89)**
Severe	**103**	**35.2**	0.96(0.72,1.26)	**182**	**51.6**	1.19(0.94,1.51)	**285**	**44.1**	1.06(0.89,1.27)
**Annual income (CNY/year)**									
≤5000	**202**	**28.4**	1.00(reference)	**378**	**52.8**	1.00(reference)	580	40.6	1.00(reference)
5000-20000	**851**	**29.9**	1.11(0.91,1.34)	**1563**	**45.8**	0.99(0.83,1.18)	2414	38.6	1.03(0.91,1.17)
>20000	**615**	**35.1**	***1.35(1.09,1.66)***	**879**	**42.8**	1.08(0.89,1.32)	1494	39.2	***1.19(1.03,1.36)***
**Sleep duration (h/d)**									
≤7	764	30.8	1.00(reference)	***1536***	***46.7***	1.00(reference)	***2300***	***39.9***	1.00(reference)
7-8	485	31.5	1.05(0.91,1.21)	***713***	***41.9***	0.99(0.88,1.13)	***1198***	***37.0***	1.01(0.92,1.11)
8-9	255	31.4	1.05(0.88,1.26)	***368***	***47.9***	***1.23(1.04,1.46)***	***623***	***39.4***	1.12(0.99,1.27)
>9	164	34.5	1.23(0.99,1.52)	***203***	***47.1***	1.23(0.99,1.52)	***367***	***40.5***	***1.18(1.02,1.37)***
**Marriage**									
No	***111***	***26.9***	1.00(reference)	***287***	***51.9***	1.00(reference)	**398**	**41.2**	1.00(reference)
Yes^b^	***1557***	***31.8***	1.18(0.93,1.51)	***2533***	***45.0***	1.21(0.99,1.47)	***4090***	***38.8***	1.10(0.95,1.28)
**Current smoking status**									
No	**783**	**34.3**	1.00(reference)	2363	45.8	1.00(reference)	**3146**	**42.3**	1.00(reference)
Yes	**885**	**29.2**	**0.78(0.69,0.89)**	457	44.5	**0.78(0.67,0.90)**	**1342**	**33.1**	**0.84(0.76,0.92)**
**Current drinking status**									
No	***941***	***32.5***	1.00(reference)	***2752***	***45.8***	1.00(reference)	**3693**	**41.5**	1.00(reference)
Yes	***727***	***30.2***	0.96(0.85,1.09)	***68***	***37.2***	0.76(0.55,1.04)	**795**	**39.7**	0.99(0.88,1.11)
**Cardiovascular disease** ^c^									
No	**1439**	**30.4**	1.00(reference)	**2139**	**42.7**	1.00(reference)	**3578**	**36.7**	1.00(reference)
Yes	**229**	**40.3**	**1.44(1.19,1.74)**	**681**	**58.0**	**1.40(1.21,1.61)**	**910**	**52.2**	**1.44(1.28,1.60)**
**Cerebrovascular disease** ^d^									
No	**1442**	**30.0**	1.00(reference)	**2459**	**43.5**	1.00(reference)	**3901**	**37.3**	1.00(reference)
Yes	**215**	**43.9**	**1.78(1.45,2.18)**	**357**	**68.4**	**1.94(1.58,2.38)**	**572**	**56.5**	**1.82(1.58,2.09)**
**Chronic kidney disease** ^e^									
No	***1609***	***31.0***	1.00(reference)	***2690***	***44.9***	1.00(reference)	**4299**	**38.5**	1.00(reference)
Yes	***30***	***46.9***	1.55(0.93,2.61)	***52***	***59.1***	1.09(0.68,1.73)	**82**	**53.9**	1.36(0.96,1.92)
**Diet score**									
<3	695	31.3	1.00(reference)	**1744**	**47.8**	1.00(reference)	**2439**	**41.6**	1.00(reference)
≥3	973	31.5	1.01(0.89,1.15)	**1076**	**42.3**	0.91(0.82,1.02)	**2049**	**36.4**	0.97(0.89,1.05)
**Beans or Bean products intake (Frequence/week)**									
Rarely	**501**	**28.4**	1.00(reference)	1251	45.4	1.00(reference)	1752	38.8	1.00(reference)
2-3times	**925**	**32.0**	***1.17(1.02,1.33)***	1262	45.3	1.03(0.92,1.16)	2221	38.7	1.09(0.99,1.18)
≥4times	**242**	**36.7**	**1.42(1.17,1.73)**	273	47.5	1.12(0.92,1.36)	515	41.7	***1.24(1.09,1.43)***
**Tea intake (Frequence/day)**									
No	788	29.7	1.00(reference)	2160	46.5	1.00(reference)	**2948**	**40.4**	1.00(reference)
rarely	458	33.4	***1.20(1.04,1.38)***	393	43.0	1.08(0.93,1.26)	**851**	**37.2**	***1.11(1.00,1.23)***
1-2 times	356	32.9	***1.21(1.03,1.42)***	245	43.4	1.03(0.85,1.24)	**601**	**36.5**	1.11(0.99,1.26)
3-4 times	66	33.2	1.18(0.86,1.63)	22	36.1	0.88(0.51,1.53)	**88**	**33.8**	1.03(0.79,1.36)

^a^Including some ethnic minorities in China, such as Mongol and Manchu.

^b^Including widowed, divorced/separated and unmarried.

^c^Cardiovascular disease include angina, myocardia infarction, arrhythmia, heart failure.

^d^Cerebrovascular disease include cerebral hemorrhage, cerebral infarction, subarachnoid hemorrhage, Transient Ischemic Attack.

^e^Chronic kidney disease nephritis, acute/chronic renal failure.

Italic and bold mean *P* <0.05; Bold only means *P* <0.001; CNY, China Yuan (1CNY = 0.161 USD); *Abbreviations:* *OR* odds ratio, *95% CI* 95% confidence interval.

### Factors associated with MetS

We conducted a multiple logistic regression analysis of MetS and its associated factors. The results are also shown in Table 2. Individuals who were women, 45 years or older, with more than one children, an annual income > 20,000 CNY/year, who slept longer (>9 hours), had cardiovascular or cerebrovascular disease, frequently consumed beans or bean products and frequently drank tea were more likely to have MetS. Current smoking status, graduating from at least middle school and taking part in moderate physical activity were found to be inversely associated with having MetS.

## Discussion

The prevalence of MetS among rural Northeast Chinese participants was estimated at 39.0% (31.4% for men and 45.6% for women) using the NCEP ATP III-modified criteria. A large proportion of participants (89.6%) exhibited at least one metabolic disorder. Of the individual criteria for MetS, elevated blood pressure (67.2%) and increased fasting glucose (47.1%) were the most common, although the rest of the criteria were also common among participants. There were significantly gender differences in the prevalence of MetS and all the metabolic disorders, except hypertriglyceridemia. Females, of older age, with more than one child, who had an annual income >20,000 CNY/year, slept longer (>9 hours), frequently consumed beans or bean products and frequently drank tea had a higher risk of having MetS, whereas those who graduated from middle school, participated in regular moderate physical activity and were current smokers had a decreased risk of having MetS.

The prevalence of MetS in our study was higher than that reported in other studies conducted in China [11–13]. Data from the China Health and Nutrition Surveys (CHNS) in 2009 showed that the prevalence of MetS was 18.2% and 10.5% using the definitions provided by the revised NCEP ATPIII and IDF, respectively [14]. WH Zhang and colleagues reported a prevalence of MetS of 25.3% in suburban Beijing during 2007–2008 [15]. Other studies of population-based studies estimated MetS prevalence among urban residents in China to be 26.04% to 32.5% in women and 17.91% to 35.1% in men using the IDF criteria [16, 17]. Of note, however, was that the prevalence of MetS among the people of rural Northeast China was higher than those in some urban areas [18]. Furthermore, the prevalence in rural China was comparable to the prevalence in developed countries, including the U.S (32.4% in women, 36.1% in men), Italy (15% in men, 18% in women) and Canada (20.5% in women, 17.8% in men) [19, 20]. The high number of residents in rural Northeast China with hypertension may be a possible explanation for the high prevalence of MetS found in our study. We found that 33.7% and 51.1% of the participants had prehypertension and hypertension, respectively, which means that less than two individuals in ten had normal blood pressure. We compared the epidemiology of hypertension in rural Northeast China to other middle- or high-income countries, such as Singapore (58.6%) and Russia (52%), and found that they have similar rates of hypertension [21, 22]. The high-salt, unhealthy diet consumed by many residents in rural Northeast China, such as commonly consumed pickled cabbage and spring onions dipped in soy sauce, elevates the blood pressure with resultant hypertension. Abdominal obesity seemed to be another important factor associated with the observed high incidence of MetS. In fact, the prevalence of MetS in our study decreased to approximately 23% when abdominal obesity was defined using a higher cut-off value, 15.1% when using WC ≥102 cm for males and WC ≥88 cm for females and 42.8% when using WC ≥90 cm for males and WC ≥80 cm for females. However, the higher cut-off value of WC likely underestimated the prevalence of abdominal obesity and MetS. In fact, our previous study reported that the prevalence of abdominal obesity was 48.6% in hypertensive women ten years ago in rural Liaoning province [23].

With regards to the prevalence of the individual metabolic disorders associated with MetS, the high prevalence of elevated blood pressure and increased fasting glucose was consistent with findings from another study of the Korean population aged >60 years, which reported a prevalence of 51.4% of participants with elevated blood pressure and 52.5% of participants with increased fasting glucose [24]. In contrast, a study held in the U.S. from 1999–2010 found that even though the incidence of MetS and related metabolic criteria had decreased, high numbers of participants with abdominal obesity (56.1%) and low HDL-C (24.3%) were still observed among Americans [25]. Similar findings were also observed in a study held in Malaysia [26]. Geographical variation and ethnic differences were relevant to the differences. We believe that demographic and socioeconomic factors may play a more important role in this difference.

In our study, we found a higher occurrence of MetS in women than in men, which was consistent with the findings of other studies [27]. Residents aged >65 years had a 1.64-fold (95% CI: 1.40, 1.91) greater risk of having MetS than those between 35–44 years old. As age advanced, individuals had greater risk of MetS because of greater risk of several other chronic diseases, which contribute to MetS, such as coronary heart disease, hypertension, diabetes and hyperlipidemia [28–30]. Our study had similar results to previous national surveys in that graduation from middle school correlated with lower risk of having MetS [31]. However, no relationship was found between those participants who had graduated from high school or higher education and the occurrence of MetS due to the small proportion of participants with a higher level of education in rural Chinese. We also found that moderate physical activity was correlated with lower prevalence of MetS, which may be due to the effect of physical activity on improving individual metabolic parameters [32]. However, vigorous physical activity did not correlate to MetS prevalence in our study because only 5.6% of participants took part in vigorous physical activity. Data showed that those who slept ≥9 hours had an increased risk for MetS, which had been observed in previous studies [33]. There was a U-shaped association between sleep duration and MetS, which indicated that both short and long self-reported sleep durations were associated with an increased risk for developing diabetes [34]. An annual income >20,000 CNY/year was correlated with an increased risk of MetS in our study; however, the relationship between income and MetS or cardiovascular risks is still controversial in both developing and developed countries [26, 35, 36].

One interesting finding in our study was that participants who had more than one child seemed have greater risk of MetS than those who had only one or did not have children. We further analyzed this finding and found that participants with one or no children were significantly younger than those with more than one child (48.7 ± 8.1 vs. 57.3 ± 10.6, *P <* 0.001). Only 3.1% of those with no or one or no children were ≥65 years old, which was significantly lower than those with more than one child (23%). Individuals with one or no children between the ages of 35–55 years made up 77.2% of the participants, while individuals who had more than one child were mostly older than 55 years (60.1%). This age-specific distribution was due to the one-child policy, which began in 1982 in China. In addition, females with more than one child had a higher incidence of MetS (50.8% vs. 37.6%, *P <* 0.001), but the same trend was not observed in males (31.3% vs. 31.5%). Previous studies have confirmed that obesity is increasing among pregnant women [37]. The more times a woman becomes pregnant, the more likely the woman is to be obese, which also increases the chance of having MetS. This difference probably warrants further study because it may imply that healthcare systems should pay attention to women who have or plan to have more than one child.

Surprisingly, smoking was correlated with a lower prevalence of MetS in rural Northeast China, while frequent consumption of beans or bean products and tea correlated with an increased risk of MetS. These results were inconsistent with the results of previous studies [38–40]. With the development of a healthy education program in rural areas over the past ten years, individuals have begun to pay attention to their health. Once participants knew they had hypertension or diabetes, they made efforts to rectify their metabolic disorders by changing their diet habits, quitting smoking and increasing their consumption of beans or bean products and tea. This change may result in a lower prevalence of smoking and increased consumption of beans and tea in individuals with MetS.

This study was performed in a representative sample of rural Chinese residents. Liaoning Province has an average population density of 297 people per square kilometer, wherein the majority of the population is settled in rural areas; thus, the findings were likely to be generally applicable to the rural Chinese population. However, some limitations of this study must be considered. First, our study was a cross-sectional study, which restricted the interpretation of the observed associations in terms of cause and effect. Longitudinal studies are required for further investigation of these findings. Second, the prevalence of MetS was based on a single assessment of blood samples, which may introduce error. In addition, although the researchers had been trained according to a standardized protocol of measurements, measurements at a single visit might lead to incorrect values for the anthropometric indexes.

## Conclusion

This study was intended to update the prevalence rates of MetS in rural Northeast China and has shown a higher prevalence of MetS in rural areas than in some urban areas in China and other countries. The associated risk factors include age, sex, physical activity, having more than one child, annual income, sleep time, consumption of beans or bean products, education status, smoking and consumption of tea. It is our hope that the high prevalence of MetS will attract the attention of the government and lead them to address this severe situation.

## Abbreviations

MetS: Metabolic syndrome
CNY: China yuan
WC: Waist circumference
BMI: Body mass index
FPG: Fasting plasma glucose
TC: Total cholesterol
LDL-C: Low-density lipoprotein cholesterol
HDL-C: High-density lipoprotein cholesterol
TG: Triglyceride
SBP: Systolic blood pressure
DBP: Diastolic blood pressure.
